# Balloon cells in type II focal cortical dysplasia: developmental origins, molecular signatures, and epileptogenic mechanisms

**DOI:** 10.3389/fcell.2026.1963535

**Published:** 2026-10-07

**Authors:** Yan Gao, Xinna Ji, Shuo Feng, Yanyan Gao, Lingxue Li, Bing Wang, Xue Zhang, Qian Chen

**Affiliations:** 1 Capital Institute of Pediatrics, Chinese Academy of Medical Sciences and Peking Union Medical College, Beijing, China; 2 Department of Neurology, Capital Center for Children’s Health, Capital Medical University, Beijing, China; 3 State Key Laboratory of Medical Molecular Biology, Institute of Basic Medical Sciences, Chinese Academy of Medical Sciences and Peking Union Medical College, Beijing, China

**Keywords:** balloon cells, epilepsy, focal cortical dysplasia, mTOR signaling, neuroinflammation, targeted therapy

## Abstract

Type II focal cortical dysplasia (FCD II) is a major cause of drug-resistant epilepsy in children, with balloon cells representing a defining histopathological feature of FCD IIb. Although diagnostically important, the biological identity and epileptogenic contribution of balloon cells remain incompletely understood. Current evidence links balloon cells to disrupted cortical development, often driven by somatic mosaic mutations in the PI3K-AKT-mTOR pathway, resulting in impaired differentiation, cellular enlargement, and hybrid neuroglial phenotypes. This review examines current evidence on the developmental origins and lineage states, molecular signatures, electrophysiological features, and network effects of balloon cells in FCD II. Persistent mTOR hyperactivation appears to interact with cytoskeletal abnormalities, oxidative stress, and local inflammatory signaling, thereby creating a microenvironment that favors epileptogenic remodeling. Despite being largely electrically silent, BCs are embedded within lesion microenvironments characterized by disrupted inhibitory circuitry, altered neurotransmitter homeostasis, and complement-associated synaptic remodeling. BCs may participate indirectly in these processes, although their cell-specific causal contribution remains unresolved. Recent single-cell and spatial transcriptomic studies further distinguish balloon cells from dysmorphic neurons and implicate them in local immune activation. Understanding balloon cell biology may reveal cell-specific therapeutic targets and support precision treatment strategies beyond surgical resection.

## 1 Introduction

Epilepsy is a common neurological disorder characterized by an imbalance between excitatory and inhibitory neurotransmission, leading to increased cortical excitability and widespread network dysfunction (Tanaka et al., 1997). Among the structural causes of drug-resistant focal epilepsy, particularly in children and young adults, focal cortical dysplasia (FCD) represents one of the most frequently encountered pathologies (Taylor et al., 1971; Blumcke et al., 2017). Originally described in its modern form by Taylor and colleagues in 1971, FCD comprises a heterogeneous group of cortical malformations. To facilitate consistent diagnosis and research, the International League Against Epilepsy (ILAE) later introduced a classification system based on reproducible neuropathological criteria (Blümcke et al., 2011; Najm et al., 2022). According to this framework, FCD Type I is characterized by isolated abnormalities of cortical lamination. In contrast, FCD Type II shows cortical dislamination together with dysmorphic neurons (FCD IIa), with balloon cells additionally present in FCD IIb. FCD Type III refers to cortical dysplasia occurring in association with a principal lesion, such as hippocampal sclerosis, tumors, or injuries acquired early in life (Najm et al., 2018; Baldassari et al., 2019).

Among these subtypes, FCD IIb is of particular biological interest because of the characteristic presence of balloon cells (BCs). BCs display a distinctive hybrid phenotype, co-expressing markers associated with both neuronal and glial lineages (Thom et al., 2007; Orlova et al., 2010). This hybrid identity suggests disrupted lineage commitment during cortical development. Owing to these striking structural features and their emerging functional significance, BCs have increasingly become a focal point for investigating the mechanisms of epileptogenesis in FCD.

In this review, we synthesize current knowledge regarding BCs in FCD IIb. We summarize their developmental origins and core biological characteristics, examine the molecular pathways driving their formation, and critically analyze their role in shaping epileptogenic circuits. By integrating these mechanistic insights, we discuss emerging targeted diagnostic and therapeutic strategies. Figure 1 summarizes the developmental origins, molecular signatures, and non-cell-autonomous network effects of BCs.

**FIGURE 1:**
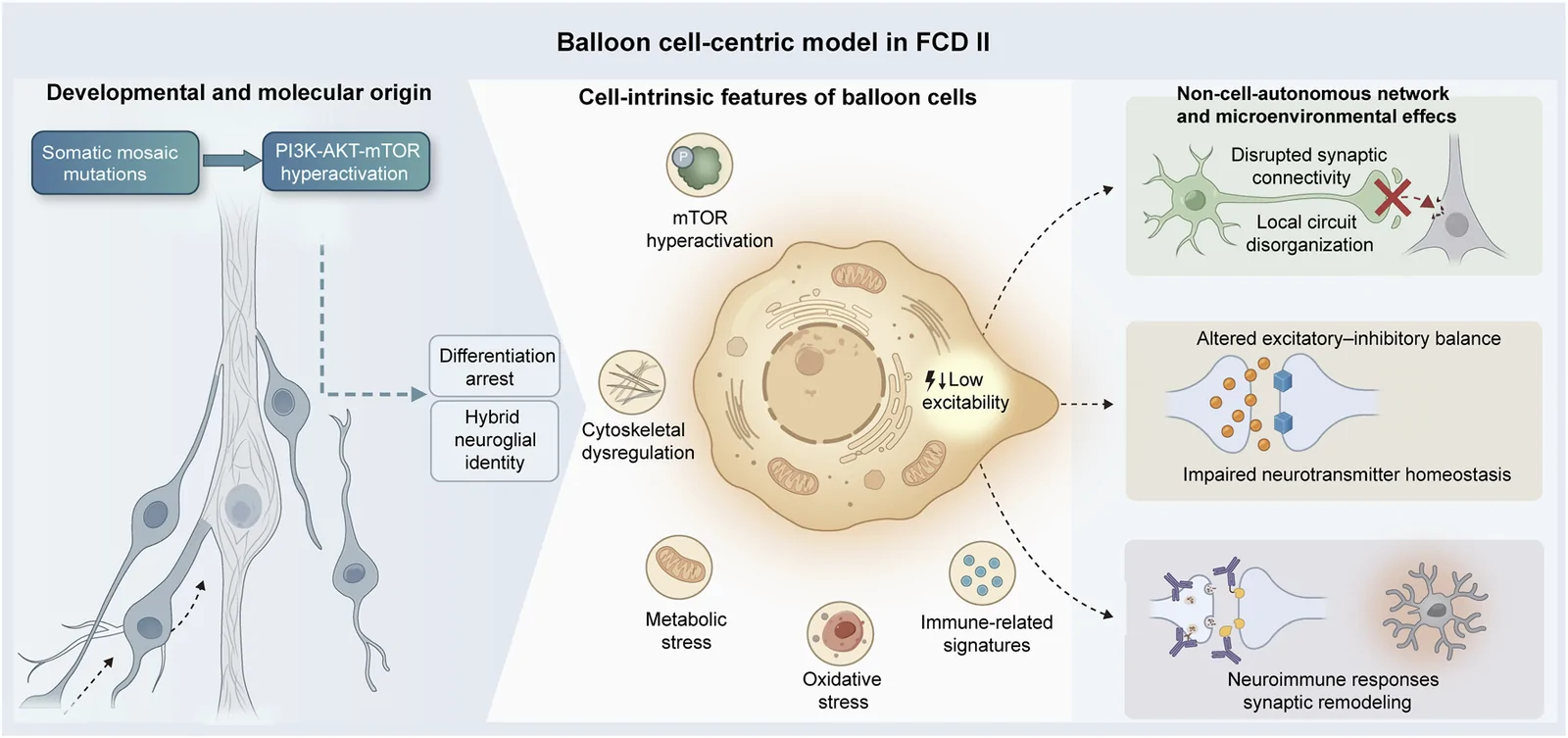
The balloon cell-centric model of epileptogenesis in type II focal cortical dysplasia (FCD II). (Left) Developmental and molecular origins: Somatic mosaic mutations drive PI3K-AKT-mTOR signaling hyperactivation in early cortical progenitors. This genetic hit is proposed to disrupt terminal differentiation and contribute to the formation of structurally enlarged balloon cells (BCs) that retain an aberrant hybrid neuroglial identity. (Middle) Cell-intrinsic features: Morphologically defined BCs often show sustained mTOR hyperactivation and cytoskeletal abnormalities, whereas oxidative-stress and immune-related signatures are also observed in BCs or BC-enriched regions. Notably, despite these severe intrinsic derangements, BCs typically exhibit low electrical excitability. (Right) Non-cell-autonomous network and microenvironmental effects: BCs and BC-enriched microenvironments are associated with disrupted synaptic connectivity and local circuit organization, altered excitatory–inhibitory balance and neurotransmitter homeostasis, and neuroimmune and synaptic remodeling. These broad network-level effects are further resolved into cell-type-specific and molecular interactions in Figure 2. Solid arrows indicate relationships supported by more direct evidence, whereas dashed arrows indicate proposed or associative interactions.

## 2 Genetic basis of type II focal cortical dysplasia and mTOR pathway dysregulation

FCD represents one of the major developmental malformations of the cerebral cortex and is a leading cause of drug-resistant focal epilepsy in children requiring surgical treatment (Crino, 2015). Among its subtypes, FCD type II has been most consistently associated with low-level somatic mosaic mutations arising during cortical development (Xu et al., 2025a). Accumulating genetic and molecular evidence indicates that FCD comprises a heterogeneous group of developmental anomalies driven by germline or somatic mutations in genes regulating the PI3K-AKT-mTOR signaling pathway, a central regulator of neuronal growth, differentiation, and migration (Iffland and Crino, 2017). These somatic mutations are thought to arise during cortical development, with the resulting cellular distribution and lesion extent influenced by when the mutation occurs and which progenitor population is affected. Human genetic and single-cell studies support the involvement of dorsal telencephalic progenitors and excitatory neurogenic lineages, although the precise progenitor population and developmental timing may vary among FCD II lesions (D'Gama et al., 2017; Chung et al., 2023). The mechanistic target of rapamycin (mTOR) signaling pathway functions as a key integrative hub that coordinates a wide range of cellular processes, including cell growth, proliferation, metabolic regulation, and autophagy, by sensing and integrating diverse intracellular and extracellular signals such as amino acid availability, growth factor signaling, hormonal cues, and cytokines (Melick and Jewell, 2020; Szwed et al., 2021). In both the developing and mature brain, mTOR signaling exerts highly context-dependent and functionally diverse effects (Lee et al., 2022). Its activity is tightly regulated by an interconnected network of upstream modulators, including positive regulators such as phosphoinositide 3-kinase (PI3K), protein kinase B (AKT), and Ras homolog enriched in brain (RHEB), as well as negative regulators such as phosphatase and tensin homolog (PTEN), the tuberous sclerosis complex (TSC), and the GAP activity toward Rags 1 (GATOR1) complex (Lee et al., 2022; Melick and Jewell, 2020). Upon activation, downstream effectors including S6 kinase 1 (S6K1) and eukaryotic translation initiation factor 4E-binding protein 1 (4EBP1), thereby promoting cap-dependent protein synthesis and other anabolic cellular processes (Szwed et al., 2021). Evidence from human surgical specimens provides direct support for sustained mTOR pathway hyperactivation in FCD II. Lim et al. demonstrated markedly elevated p-S6 expression in FCD II tissues harboring somatic TSC1 or TSC2 mutations, and p-S6-positive neurons showed increased cell volumes compared with normal neurons (Lim et al., 2017). Beyond abnormal cellular growth, FCD II is also characterized by dysregulation of apoptotic pathways. Mitochondrial apoptosis is regulated by the balance between anti-apoptotic proteins such as B-cell lymphoma 2 (Bcl-2) and pro-apoptotic factors including Bax. In balloon cells, upregulated Bcl-2 expression has been reported, suggesting reduced susceptibility to apoptosis and prolonged survival of aberrant cell populations within the dysplastic cortex (Ying et al., 2005).

The process of normal cortical development provides valuable insights into the biological identity of balloon cells. Following neural tube formation, radial glial cells within the ventricular zone act as neural progenitors and provide a scaffold for the migration of newly generated neurons toward the cortical plate (Noctor et al., 2001). Through direct and indirect neurogenic divisions, including the generation of intermediate progenitor cells that proliferate in the subventricular zone, radial glia contribute to the formation of the layered cerebral cortex (Noctor et al., 2004; Haubensak et al., 2004; Miyata et al., 2004; Englund et al., 2005). More recent studies have further highlighted the developmental importance of the outer subventricular zone and discussed these developmental principles in relation to abnormal non-neuronal cells in FCD IIb and related mTORopathies (Zarzor et al., 2023; Zhang et al., 2024). Disruption of this tightly regulated developmental program is thought to underlie balloon cell formation, as their mixed neuroglial phenotype is consistent with arrested differentiation of radial glial-derived progenitors. Structural and ultrastructural studies further support a developmental origin for these abnormalities. Using ultra-high-field 7 T MRI in combination with detailed histological and ultrastructural analyses of resected FCD II specimens, Zucca et al. demonstrated that lesion cores are characterized by focal accumulations of abnormal cells and severely disorganized fiber architecture, corresponding to regions of intracortical signal inhomogeneity (Zucca et al., 2016). In FCD IIb, prominent white matter hyperintensity was associated with defective myelination, substantial axonal loss, and the presence of aberrant cells. These findings suggest that white matter alterations in FCD IIb reflect impaired myelin formation and delayed maturation, potentially driven by the excessive presence of balloon cells during early corticogenesis (Zucca et al., 2016). Balloon cells also exhibit features consistent with cell-cycle arrest and premature cellular senescence (Ribierre et al., 2024), indicating broader disturbances in developmental and homeostatic regulation. Ribierre et al., through electron microscopic analysis of surgically resected FCD II specimens carrying pathogenic mTOR-activating variants, identified aggregates of enlarged, electron-dense lysosomes at various stages of maturation, along with abundant multivesicular bodies in both dysmorphic neurons and balloon cells—ultrastructural features commonly associated with senescent cell phenotypes. Importantly, these abnormalities were absent in neighboring morphologically normal neurons, underscoring their specificity to pathologically altered cell populations (Ribierre et al., 2024). However, due to the lack of fetal cortical tissue from human FCD II cases, it remains challenging to determine whether these senescent-like features arise during early proliferative stages of corticogenesis or develop later in postnatal, post-mitotic neurons.

## 3 Biological characteristics of balloon cells

### 3.1 Morphological, molecular, and developmental identity of balloon cells

BCs constitute a hallmark pathological cell population in FCD IIb and exhibit distinct morphological, molecular, and developmental abnormalities that are closely associated with dysregulated mTOR signaling. Beyond their diagnostic morphology, BCs show structural abnormalities consistent with incomplete neuronal differentiation, including reduced process formation and abnormal cytoplasmic organization. Compared with FCD IIa, both typical and dysmorphic neurons in FCD IIb demonstrate more severe dendritic and spine abnormalities. These abnormalities include reduced dendritic arborization, spine depletion, altered spine morphology, and the presence of multiple dendritic varicosities within specific neuronal populations (Rossini et al., 2021; Zhang et al., 2024). These observations indicate that the presence of BCs is associated not only with glial lineage abnormalities but also with profound impairment of neuronal structural integrity. Ultrastructural analyses further refine this morphological profile. Electron microscopy has revealed that BCs contain abundant intermediate filaments and numerous mitochondria concentrated within the central cytoplasm. Neurosecretory granules were not observed (Yasin et al., 2010). The overall cellular architecture was largely preserved despite marked cytoplasmic enlargement. These ultrastructural observations indicate abnormal cytoplasmic organization, but they do not by themselves establish a specific mechanism of developmental arrest or impaired intracellular trafficking.

Genetic studies have demonstrated that dysmorphic neurons and balloon cells can harbor the same pathogenic mTOR-pathway somatic variants within individual FCD II lesions. These observations support, but do not directly establish, the possibility that at least some BCs and DNs originate from an early mutational event affecting a progenitor with multilineage potential (Baldassari et al., 2025; Baldassari et al., 2019). These mutations result in pathological mTOR hyperactivation, leading to defective autophagy, abnormal cell growth, and impaired differentiation (Marsan and Baulac, 2018). Consistent with this, downstream mTOR complex 1 (mTORC1) effectors, including p-S6 and 4EBP1, are markedly elevated in BCs, directly reflecting sustained pathway activation. Nevertheless, despite these shared genetic alterations, the precise and potentially distinct contributions of dysmorphic neurons and balloon cells to epileptogenesis remain incompletely understood. A central unresolved question is whether they represent fundamentally distinct cell types or alternative phenotypic states arising along a common developmental trajectory.

According to the ILAE classification of focal cortical dysplasia, balloon cells are defined by their enlarged cell morphology, opalescent eosinophilic glassy cytoplasm lacking Nissl substance, and frequent multinucleation (Blümcke et al., 2011). They also exhibit a hybrid phenotype characterized by the co-expression of neuronal and glial markers, consistent with an incomplete lineage commitment. BCs express markers associated with neural progenitors or neuroglial precursors, including nestin, vimentin, SRY-box transcription factor 2 (Sox2), cluster of differentiation 34 (CD34), Krüppel-like factor 4 (Klf4), c-Myc, collapsin response mediator protein 4 (CRMP4), and glial fibrillary acidic protein (GFAP) δ (Oh et al., 2008; Taylor et al., 2001; Lamparello et al., 2007; Orlova et al., 2010). Although BCs express cell cycle-associated proteins such as minichromosome maintenance complex component 2 (Mcm2) and cluster of differentiation 133 (CD133), they lack canonical proliferation markers, including Ki-67 and proliferating cell nuclear antigen (PCNA), indicating arrest at the G1/S checkpoint in the absence of active mitosis (Iffland and Crino, 2017). This molecular profile supports a phenotype resembling non-dividing, developmentally arrested stem-like cells rather than neoplastic transformation.

### 3.2 Cellular origin and lineage specification

The cellular origin of balloon cells has been a longstanding subject of debate. A pivotal study by D’Gama et al. applied single-cell nuclear genotyping in FCD tissue harboring known pathogenic variants. In lesions with low mutant allele frequencies, pathogenic mutations were largely restricted to neurons, suggesting origin from neuron-committed progenitors. In contrast, lesions with more extensive involvement exhibited mutations in both neuronal and glial populations, consistent with an earlier mutational event occurring in a multipotent progenitor capable of generating both lineages (D'Gama et al., 2017). These findings are consistent with the hypothesis that at least a subset of balloon cells may arise from incompletely differentiated progenitor populations carrying mTOR pathway-activating mutations; however, they do not establish the lineage relationship of human BCs directly. Aberrant mTOR signaling disrupts normal programs of differentiation and migration, ultimately giving rise to enlarged, malformed cells with mixed neuronal and glial characteristics. Additional support for this lineage model comes from molecular profiling studies. Shen et al. reported robust expression of vascular endothelial growth factor C (VEGF-C) and its receptors VEGFR-2 and VEGFR-3 specifically in balloon cells within FCD IIb lesions. Importantly, VEGF-C expression co-localized with vimentin, an intermediate filament marker associated with neural progenitors and radial glial cells, and VEGFR-2 was also enriched in vimentin-positive BCs, whereas neither VEGF-C nor its receptors co-localized with the neuronal marker NeuN (Shen et al., 2022). This expression pattern suggests that BCs may arise from neural precursor cells that undergo developmental arrest and maintain their pathological phenotype through VEGF-C–mediated autocrine or paracrine signaling. Experimental models provide additional, although indirect, support for a developmental link between focal mTORC1 dysregulation and the generation of enlarged abnormal cells with BC-like features. Ribierre et al. generated a focal chimeric Depdc5 knockout mouse model that reproduced several FCD-associated features, including focal mTORC1 hyperactivation, cortical dysplasia-like abnormalities, neuronal hypertrophy, balloon-like cells, and spontaneous seizures (Ribierre et al., 2018). These findings support a causal role for focal mTORC1 dysregulation in FCD-like pathology and the generation of enlarged abnormal cells. However, this model does not by itself establish the developmental lineage of human BCs.

### 3.3 Electrophysiological properties of balloon cells

Available recordings from morphologically identified BCs indicate that these cells are generally non-excitable under the experimental conditions studied. BCs do not generate action potentials, lack detectable spontaneous synaptic events, and show no detectable response to glutamate, arguing against a direct role as primary electrical generators (Cepeda et al., 2003). However, these findings do not exclude lesion-level effects associated with BC-enriched regions.

Accordingly, BCs are unlikely to act as direct cellular generators of epileptiform discharges. Their possible contribution to epileptogenesis should instead be considered at the level of the surrounding dysplastic tissue and local cellular microenvironment. However, direct electrophysiological evidence linking BCs to altered synaptic transmission or neurotransmitter clearance remains limited.

### 3.4 Developmental timing of balloon cells

BC formation is generally considered to reflect disturbances arising during prenatal corticogenesis, although the precise developmental timing and progenitor population giving rise to human BCs remain unresolved. A proposed radial glial/progenitor origin is consistent with the aberrant expression of neural progenitor and radial glial markers, including vimentin and SOX2, together with incomplete or mixed lineage-associated phenotypes (Lamparello et al., 2007). Recent multi-omic and single-nucleus analyses have implicated dividing radial glia and their daughter excitatory neurons as developmentally relevant cellular contexts for mTOR-related malformations of cortical development (Chung et al., 2023). Within this proposed developmental framework, an early mTOR-activating event may disturb normal differentiation and lead to the persistence of an immature or mixed-lineage phenotype similar to that seen in BCs (Lamparello et al., 2007; D'Gama et al., 2017; Chung et al., 2023).

## 4 Balloon cells and epileptogenesis

Building on the framework summarized in Figure 1, this section examines how BCs and BC-enriched microenvironments interact with surrounding neuronal and glial networks, as illustrated in Figure 2.

**FIGURE 2:**
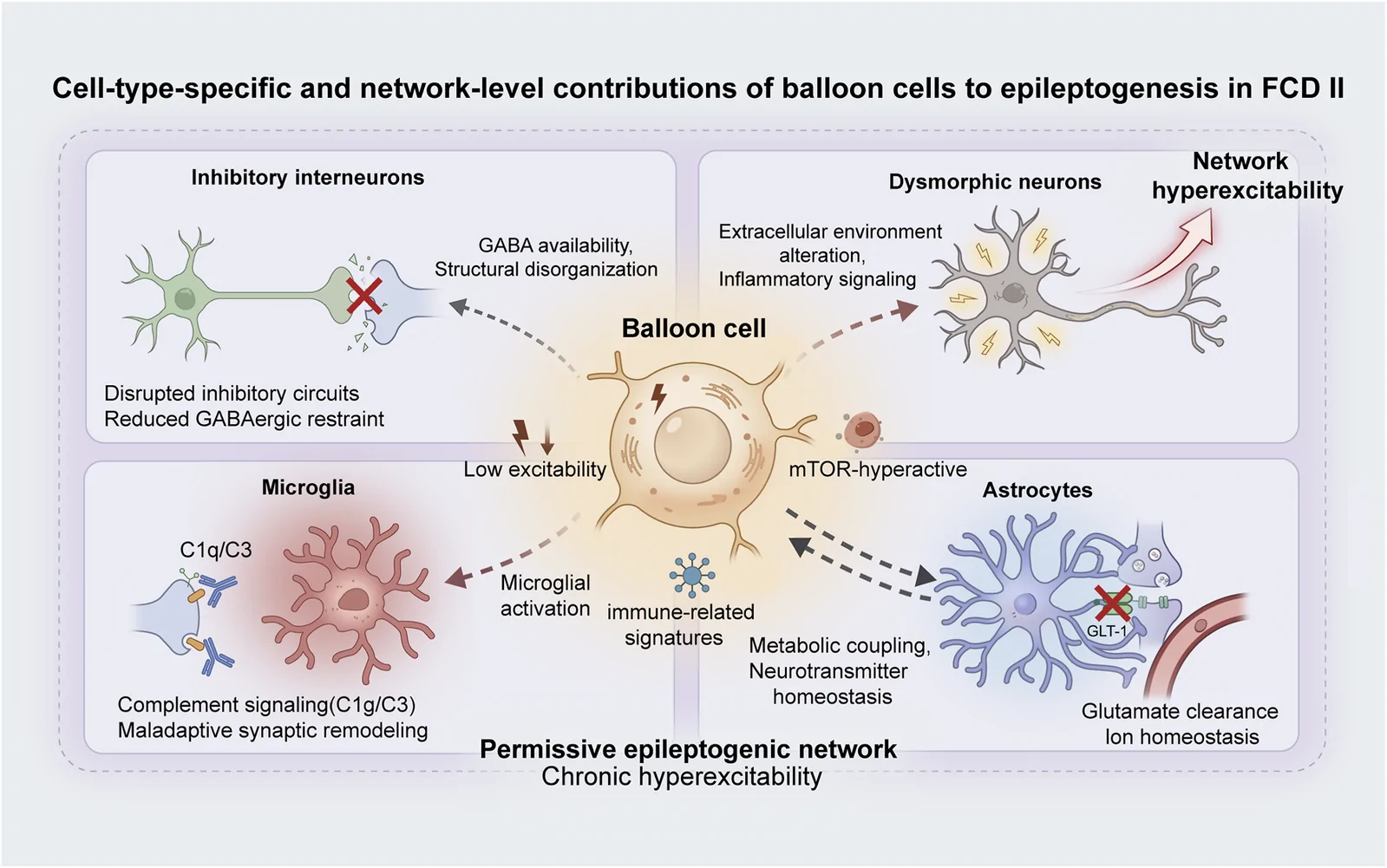
Cell-type-specific and network-level contributions of balloon cells to FCD II epileptogenesis. Schematic representation of the complex crosstalk between the central balloon cell (BC) and surrounding cellular populations. This figure expands on the broader network-level effects summarized in Figure 1 by illustrating the corresponding cell-type-specific interactions within the FCD IIb microenvironment. Despite their low intrinsic excitability, BCs are embedded within a permissive epileptogenic microenvironment involving inhibitory interneurons, dysmorphic neurons, astrocytes, and microglia. Inhibitory interneurons: BC-enriched lesions exhibit disrupted inhibitory circuitry and reduced GABAergic restraint together with structural disorganization of synapses (indicated by the red cross). Dysmorphic neurons (DNs): Inflammatory and extracellular microenvironmental alterations may facilitate hyperexcitability in adjacent DNs, for which experimental studies provide more direct functional evidence of a contribution to epileptiform activity than is currently available for BCs. Astrocytes: Altered metabolic coupling may contribute to impaired neurotransmitter and ion homeostasis, notably characterized by defective glutamate clearance (highlighted by the downregulation/loss of GLT-1 transporters). Microglia: BC-enriched regions are associated with microglial activation and complement-related synaptic remodeling involving classical complement components such as C1q and C3; whether BCs initiate or sustain this process remains unresolved. Dashed arrows indicate proposed or associative interactions, whereas solid arrows denote relationships supported by more direct functional evidence.

### 4.1 Structural and developmental context of balloon cells in FCD IIb

Histopathologically, FCD II is defined by cortical disorganization and dysmorphic neurons, with balloon cells additionally present in type IIb (Lamberink et al., 2020). Beyond these hallmark cellular abnormalities, FCD IIb is characterized by a constellation of architectural alterations, including abnormal myelination, distorted oligodendrocyte morphology, cortical thickening, and an indistinct gray-white matter boundary (Scholl et al., 2017; Wang et al., 2023). These features establish the structural substrate upon which epileptogenic networks emerge. A defining feature of FCD IIb is the heterogeneous spatial distribution of BCs, which varies substantially across patients. BCs often display a focal or patchy arrangement and may intermix with dysmorphic neurons (DNs) within individual lesions. Topographic analyses consistently demonstrate a preferential accumulation of BCs within the subcortical white matter and along the gray-white matter junction, extending radially toward the cortex (Rossini et al., 2017b). This distribution pattern is consistent with abnormal radial migration or arrested differentiation during corticogenesis and is thought to contribute directly to cortical dyslamination and the blurred gray-white matter interface characteristic of FCD IIb. Neuroimaging findings closely mirror these histopathological observations. Magnetic Resonance Imaging (MRI) typically reveals cortical thickening, abnormal sulcal and gyral morphology, subcortical white matter hyperintensities, and the transmantle sign, a funnel- or column-shaped region of increased T2/FLAIR signal intensity extending from the ventricular zone to the cortical surface (Zhang et al., 2024; Kimura et al., 2019). This radiological hallmark correlates strongly with the radial accumulation of BCs and is associated with reduced myelinated fiber density, increased BC burden, and a reduced number of oligodendrocytes with abnormal nuclear morphology (Bartolini et al., 2019). These features collectively suggest that disrupted myelination in FCD IIb may be associated with abnormal cellular differentiation and maturation during prenatal cortical development. Moreover, the transmantle sign implicates dysfunction of radial glial progenitor cells (Zhang et al., 2024), which normally provide the scaffold for neuronal migration from the periventricular germinal zone to the developing cortex (Noctor et al., 2001). Disruption of this scaffold likely contributes to aberrant cell positioning, cortical dyslamination, and the BC-enriched radial corridor visualized on MRI. The interactions between BCs and surrounding neuronal and glial populations, and their role in driving network-level hyperexcitability in FCD II, are summarized in Figure 2.

The precise ontogeny of DNs and BCs remains debated. A proposed developmental model suggests that earlier mTOR-pathway mutations affecting multipotent dorsal telencephalic progenitors may generate mutant cells across both neuronal and glial lineages, potentially giving rise to both BCs and DNs (D'Gama et al., 2017; Baldassari et al., 2019; Chung et al., 2023). In contrast, mutations occurring later in more lineage-restricted excitatory progenitors may mainly give rise to neuronal abnormalities such as DNs (D'Gama et al., 2017; Chung et al., 2023). This interpretation is also consistent with the immature and mixed-lineage molecular phenotype of BCs; however, marker expression alone does not directly establish their progenitor of origin (Lamparello et al., 2007). The relationship between mutation timing, progenitor identity, and the development of BCs or DNs should therefore be considered a proposed model rather than an established lineage relationship in human FCD. Furthermore, patient-derived forebrain organoids have reproduced several pathological features of FCD II, including BC- and DN-like abnormal cells, and provide experimental support for altered cell-fate specification (Xu et al., 2025a). However, these *in vitro* models do not reproduce the full epileptogenic network of human FCD and cannot establish a direct lineage relationship between BCs and DNs. Their findings instead provide experimental support for abnormal developmental pathways in FCD.

### 4.2 Indirect contributions of balloon cells to network excitability

BCs have traditionally been regarded as electrically silent, based on the absence of spontaneous action potentials in brain slices enriched with these cells (Cepeda et al., 2003). While BCs do not appear to generate action potentials autonomously, recent clinicopathological correlation studies have demonstrated that BC-rich regions are associated with specific rhythmic epileptiform discharges (REDs) (Xu et al., 2025b). This observation indicates that BC-rich regions are associated with epileptogenic network activity, rather than implicating BC intrinsic excitability. However, this spatial association does not establish BCs as the causal source of these discharges. The apparent electrical silence of BCs does not preclude their association with cortical network abnormalities. FCD IIb lesions exhibit abnormalities in extracellular ion regulation, glutamate handling, and inhibitory neurotransmission. These alterations are likely mediated predominantly by dysmorphic neurons, reactive astrocytes, inhibitory interneurons, and other components of the dysplastic tissue rather than by the intrinsic electrical activity of BCs. Collectively, these lesion-level abnormalities may create a permissive environment for hypersynchronous discharges, even though BCs themselves do not generate action potentials (Aronica and Crino, 2014). Moreover, BC-enriched regions exhibit structural disorganization, inflammatory signaling, and altered expression of growth factors and extracellular matrix-related molecules. These lesion-level abnormalities may influence local circuit organization and glial responses, although their direct attribution to BCs remains uncertain. These mechanisms may help explain the association between BC enrichment and REDs despite the lack of intrinsic electrical activity.

The available functional evidence differs between BCs and DNs. Direct electrophysiological recordings from morphologically identified BCs show low intrinsic excitability, absence of action potential generation, and little or no detectable spontaneous synaptic activity, suggesting that BCs are unlikely to act as direct electrical generators (Cepeda et al., 2003). IIn contrast, stronger functional evidence is available for DNs. In an FCD II mouse model, selective ablation of DNs suppressed established seizures and normalized abnormalities in the surrounding network, supporting a direct role for DNs in seizure activity in this model (Xu et al., 2025c). Similar intervention studies targeting BCs have not yet been reported. Current evidence therefore provides stronger support for a direct contribution of DNs to epileptiform activity, whereas the role of BCs is supported mainly by indirect evidence from the surrounding lesion microenvironment.

### 4.3 mTOR hyperactivation as a central driver of epileptogenic network remodeling

*In vivo* evidence from Lim et al. demonstrates that even a small proportion of cells harboring Tsc1 or Tsc2 loss-of-function mutations, and therefore exhibiting mTOR hyperactivation, is sufficient to induce spontaneous behavioral seizures and ictal discharges in mouse models (Lim et al., 2017). This finding shows that a small population of mTOR-dysregulated cells can be sufficient to induce seizures in an experimental model; however, it does not establish the relative causal contributions of human BCs and DNs. Thus, although balloon cells themselves may not directly generate epileptiform activity, mTOR dysregulation across dysplastic cell populations may reshape the local cortical environment. Beyond the developmental effects described above (Park et al., 2018), mTOR activation alters cell growth, metabolism, and signaling within the lesion. Downstream effectors such as p-S6K1 and 4EBP1 enhance cap-dependent translation and lipid biosynthesis, driving cytomegaly and metabolic reprogramming characteristic of BCs. At the network level, mTOR activation should therefore be viewed not only as a driver of cellular enlargement, but also as an upstream organizer of local circuit remodeling, glial activation, and altered inhibitory signaling.

Consistent with this view, Zhong et al. examined inhibitory circuitry in an experimental model of FCD driven by somatic mTOR hyperactivation. They reported an approximately 20%–25% reduction in GABAergic interneuron density and impaired inhibitory synaptic transmission onto mTOR-hyperactivated dysmorphic neurons (Zhong et al., 2021). These findings support impaired inhibitory input to dysmorphic neurons in that model; they do not demonstrate mIPSC abnormalities in BCs. Whether BCs contribute indirectly to inhibitory network remodeling remains unresolved and requires cell-type-specific investigation.

### 4.4 Neuroimmune activation and complement-mediated synaptic remodeling

Persistent neuroinflammation is a consistent pathological feature of FCD IIb and is increasingly recognized as a key contributor to epileptogenesis (Vezzani et al., 2019). Elevated levels of inflammatory mediators, including HMGB1, TLR4, IL-1β, TNF-α, and IL-6 (Huang et al., 2022), reflect widespread activation of innate immune pathways. Activated microglia and astrocytes, together with neurons and endothelial cells of the blood-brain barrier, constitute the principal cellular mediators of this inflammatory response (Vezzani et al., 2019). Zimmer et al. demonstrated that FCD IIb elicits a particularly robust inflammatory response, characterized by upregulated C-C motif chemokine ligand 2 (CCL2) and human leukocyte antigen class II (HLA-II) expression in microglia surrounding BCs (Zimmer et al., 2021). These findings suggest that BCs may participate in microglial recruitment, chemokine signaling, and antigen-presentation-related immune activation. This inflammatory role may also explain why FCD IIa, which lacks BCs, exhibits comparatively milder inflammatory profiles. Complement-mediated mechanisms further amplify neuroinflammatory remodeling. Rossini et al. showed that reactivation of complement-dependent synaptic pruning contributes to the marked dendritic spine loss observed in FCD IIb. Complement components such as C1q, C3b, iC3b, and C3c are selectively enriched in the dysplastic core of FCD IIb lesions but absent in perilesional cortex and other epilepsy types (Rossini et al., 2023). This pattern suggests that complement activation is intrinsic to FCD IIb pathology rather than a secondary consequence of chronic seizures (Rossini et al., 2017a; Rossini et al., 2023). As proposed by Zimmer et al., BCs, immature, hybrid glioneuronal cells uniquely present in FCD IIb, may serve as contributors to local immune activation (Zimmer et al., 2021). Within this inflammatory and complement-active microenvironment, excessive microglial engulfment of synapses and dendritic spines may contribute to pathological network remodeling and cortical hyperexcitability. Complement signaling is physiologically involved in synaptic refinement during central nervous system (CNS) development, with C1q and C3 participating in the elimination of developing synaptic inputs and microglia mediating complement-dependent engulfment (Stevens et al., 2007; Schafer et al., 2012). In FCD IIb, this developmental pathway appears aberrantly re-engaged and is associated with dendritic spine loss and synaptic remodeling (Rossini et al., 2023). Thus, modulating inflammatory mechanisms may offer therapeutic potential in the management of FCD IIb.

### 4.5 BC-associated molecular regulatory pathways beyond mTOR

Beyond canonical mTOR signaling, multiple molecular alterations have been reported in morphologically identified BCs, DNs, BC-enriched regions, or whole FCD IIb lesions. These findings implicate pathways involved in neuronal morphogenesis, synaptic organization, and neuroimmune signaling, but their direct attribution to BCs varies substantially among studies. Emerging evidence implicates aberrant cluster of differentiation 47 (CD47)/signal regulatory protein α (SIRP-α), Plexin-B2/semaphorin 4C (Sema4C), and leukocyte immunoglobulin-like receptor subfamily B member 2 (LILRB2) signaling in FCD IIb-associated developmental, synaptic, and neuroimmune alterations. CD47, widely expressed in the CNS, mediates inhibitory signaling through its receptor SIRP-α on neurons and microglia (Sun et al., 2016). Sun et al. reported significantly reduced CD47 and SIRP-α expression in BCs and DNs within FCD IIb lesions. Given that CD47/SIRP-α signaling promotes neurite outgrowth and dendritic spine formation (Sun et al., 2016), its deficiency may contribute to disrupted neuronal migration and differentiation (Yasin et al., 2010). Concurrently, insufficient CD47/SIRP-α signaling removes inhibitory constraints on microglial activation. Under physiological conditions, neuronal CD47 engagement suppresses microglial phagocytic reactivity; thus, its downregulation enhances microglial activation, a hallmark of the FCD IIb inflammatory microenvironment (Marchi et al., 2014; Sun et al., 2016). This aberrant neuroimmune cross-talk may amplify local inflammation, sustain complement activation, and promote chronic neuronal instability, further aggravating epileptogenicity. Dai et al. identified abnormal expression of Plexin-B2 and its ligand Sema4C in cortical lesions from patients with FCD IIb, with increased Plexin-B2 expression and altered cellular distribution of both Plexin-B2 and Sema4C (Dai et al., 2024). Plexin-B2 was prominently expressed in BCs, whereas Sema4C expression was reduced in abnormal cells, including BCs and DNs (Dai et al., 2024). Independent experimental studies have also implicated Plexin-B signaling in excitatory and inhibitory synapse development (McDermott et al., 2018), and elevated Plexin-B2 expression correlated with increased seizure frequency in patients with FCD IIb (Dai et al., 2024). The heterogeneous expression of the Plexin-B2/Sema4C system across dysplastic cell populations suggests phenotypic immaturity and altered synaptic organization that may contribute to hyperconnected or miswired microcircuits (Dai et al., 2023; McDermott et al., 2018; Dai et al., 2024). Human LILRB2 is a regulator of synaptic plasticity, neurite growth, and inflammatory responses, indicating its potential involvement in the pathogenesis of epilepsy. Yue et al. demonstrated increased expression of LILRB2 and downstream effectors (POSH, SHROOM3, ROCK1/2) in the cerebral cortex of children with FCD type IIb, with LILRB2 levels inversely correlated with seizure frequency (Yue et al., 2019). Co-localization of LILRB2 with neuronal and astrocytic markers suggests roles in both excitatory synaptic modulation and glial-mediated inflammation. The inverse correlation between LILRB2 expression and seizure frequency argues against a simple pro-epileptogenic interpretation. Increased LILRB2 signaling may represent a compensatory, context-dependent, or disease-stage-dependent response rather than a direct driver of seizure activity. Whether LILRB2 signaling exerts compensatory, neuroprotective, or pro-inflammatory effects in different cellular compartments remains unresolved and warrants further mechanistic investigation.

### 4.6 Integrative insights from spatial transcriptomics

To integrate mechanistic insights across experimental systems, Figure 3 summarizes the hierarchical levels of evidence supporting BC-associated pathogenic mechanisms in FCD II. Spatial transcriptomic analyses by Wang et al. of fresh-frozen FCD IIb tissue revealed distinct transcriptional signatures in DN- and BC-enriched regions (Wang et al., 2024). Importantly, these regions represent local microenvironments enriched for DNs or BCs, rather than purified single-cell populations. DN-enriched regions exhibit transcriptional signatures associated with altered membrane excitability and seizure initiation, with enrichment of mTOR signaling, autophagy, ubiquitin–proteasome-related pathways, and regulation of membrane potential (Wang et al., 2024). These findings suggest dysregulated proteostasis and membrane potential regulation in DN-enriched regions. In contrast, BC-enriched regions displayed transcriptomic features dominated by immune activation, consistent with enhanced neuroinflammatory responses and complement cascade engagement. Wang et al. further validated increased expression of complement-associated proteins, including C3 and clusterin, in BCs by immunohistochemistry (Wang et al., 2024). Despite inter-individual variability, the convergence of immune signatures suggests that BC-enriched microenvironments may contribute to local inflammatory remodeling in FCD IIb. These spatial transcriptomic findings should be distinguished from the bulk transcriptomic and immunohistochemical evidence reported by Zimmer et al. Comparative immune profiling demonstrated greater activation of innate and adaptive immune pathways in FCD IIb than in FCD IIa, with increased HLA class I and II expression and enhanced T-cell infiltration (Zimmer et al., 2021). Notably, antigen presentation levels correlate with BC density, supporting the view that at least a subset of BCs, or BC-enriched regions, may contribute to immune activation in FCD IIb (Zimmer et al., 2021). These findings provide complementary but distinct levels of evidence, linking BC-enriched microenvironments to inflammatory and complement-related remodeling in FCD IIb.

**FIGURE 3:**
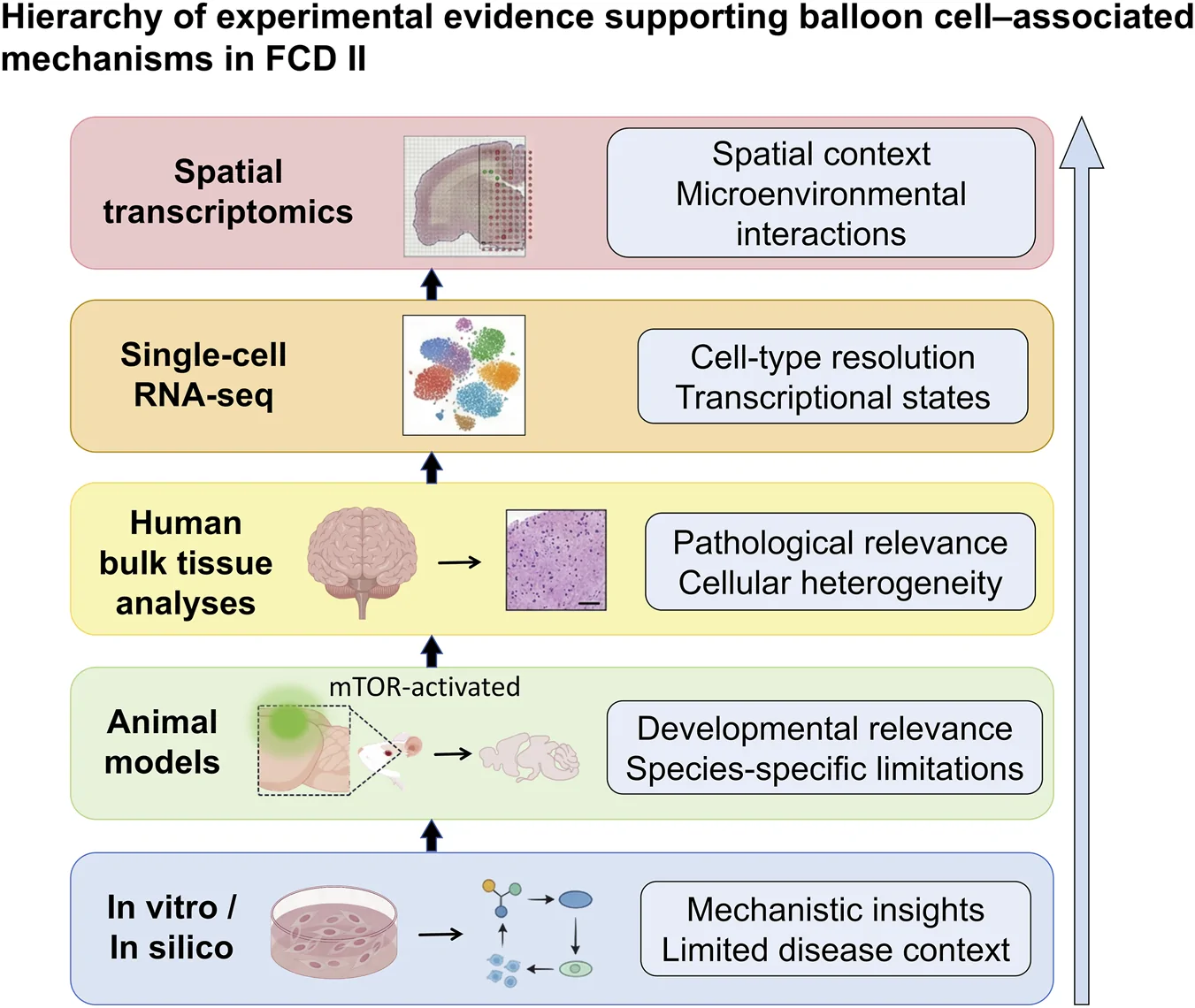
Hierarchy of experimental evidence supporting balloon cell-associated mechanisms in FCD II. Schematic overview illustrating the multi-tiered experimental approaches used to elucidate balloon cell (BC) pathology. The platforms are arranged from bottom to top (indicated by the upward arrow) reflecting increasing cellular resolution and human microenvironmental context. *In vitro*/In silico models: Provide foundational mechanistic insights into signaling pathways (e.g., mTOR cascade) but intrinsically lack the complex architectural context of the human epileptic brain. Animal models: Those utilizing targeted mTOR hyperactivation can recapitulate key developmental features such as cortical malformations and cytomegaly, yet remain constrained by species-specific developmental divergences. Human bulk tissue analyses: Utilize surgically resected FCD tissue to ensure direct clinical and pathological relevance, though interpretations are frequently confounded by bulk cellular heterogeneity. Single-cell RNA-seq (scRNA-seq): Addresses bulk-tissue limitations by enabling cell-type-resolved characterization of transcriptional states associated with BCs and DNs. Spatial transcriptomics: Provides region-resolved transcriptomic information by mapping gene expression profiles onto their anatomical coordinates, thereby revealing DN-enriched and BC-enriched microenvironments and associated immune-related signatures within the dysplastic cortex.

Because the underlying pathology strongly influences surgical outcome in focal epilepsy, a comprehensive understanding of both the pathophysiological mechanisms and disease trajectory is essential (Wang et al., 2023). Several molecular pathways implicated in FCD IIb remain incompletely characterized, underscoring the need for more systematic and mechanistic studies.

## 5 Advances in balloon cell–based diagnosis and molecular characterization

### 5.1 Cellular and epigenetic foundations of FCD IIb diagnosis

Since the initial description of FCD, disruption of the normal cellular architecture of the cerebral cortex has been recognized as both a defining pathological feature and a core diagnostic criterion. Although disease severity and histopathological subtypes vary, FCD lesions typically manifest as either microscopic or macroscopically detectable abnormalities upon neuropathological examination (Iffland and Crino, 2017). These architectural disturbances reflect perturbations occurring during cortical development and provide the structural substrate for the electrophysiological instability characteristic of FCD-associated epileptogenic zones. A recent study systematically characterized layer-specific neuronal subtypes and alterations in cortical neuronal composition in FCD IIb using an extensive panel of layer-selective markers (Nakagawa et al., 2017). Surprisingly, even in severe cases of FCD IIb, overall cellular diversity and the fundamental framework of cortical lamination were largely preserved (Nakagawa et al., 2017). This observation suggests that gross laminar organization remains intact and that pathological changes may preferentially affect specific neuronal subpopulations rather than globally disrupting cortical layering. Consistent with this notion, quantitative analyses revealed a reduced density of parvalbumin (PV)-immunoreactive interneurons, together with a concomitant increase in TLE4-immunoreactive neurons at the gray–white matter junction, indicating disproportionate alterations in deeper cortical layers (Nakagawa et al., 2017). Given the critical role of PV-positive interneurons in synchronizing cortical oscillations and maintaining excitatory–inhibitory balance, a reduction in PV-mediated inhibitory control may facilitate local network hyperexcitability and increase seizure susceptibility in FCD IIb.

Beyond histoarchitectural abnormalities, epigenetic profiling has emerged as a powerful tool for refining the molecular classification of FCD. Kobow et al. were the first to demonstrate that genome-wide DNA methylation signatures can reliably distinguish FCD subtypes and differentiate them from both temporal lobe epilepsy and non-epileptic control tissue (Kobow et al., 2019). These findings provide strong epigenetic evidence supporting FCD IIb as a distinct molecular entity and identify potential biomarkers for diagnostic stratification. Notably, differentially methylated regions in FCD are predominantly enriched within intergenic regions, genomic deserts, and enhancer elements rather than classical promoter regions. This epigenomic pattern differs markedly from that observed in brain tumors and underscores a pathogenic mechanism centered on dysregulation of long-range gene regulatory elements. Enhancer- and intergenic-region methylation is particularly relevant in neurodevelopmental disorders, as it governs chromatin accessibility, lineage-specific transcriptional programs, and spatiotemporal gene regulation (Kobow et al., 2019; Li et al., 2018). Accordingly, these findings suggest that FCD IIb pathogenesis involves widespread disruption of epigenomic regulatory architecture rather than isolated promoter-specific changes. Although a subset of FCD cases harbor mutations in mTOR pathway genes (e.g., DEPDC5, AKT1), whole-genome methylation analyses indicate that mTOR-related loci are not the primary targets of methylation alterations. Instead, Kobow and colleagues identified a significant inverse correlation between DNA methylation and gene expression, supporting a model in which epigenetic dysregulation modulates transcriptomic landscapes independently of, or synergistically with, underlying genetic mutations (Kobow et al., 2019). In this context, the characteristic methylation profile of FCD IIb may influence balloon cell properties and network hyperexcitability through broad transcriptional reprogramming. These data indicate that FCD IIb arises from a complex interplay between genetic lesions and epigenetic remodeling, with mTOR pathway mutations providing an initial molecular trigger and enhancer-driven epigenomic alterations shaping disease progression.

### 5.2 Transcriptomic and molecular biomarkers associated with balloon cells

A multicenter transcriptomic study analyzing FCD II subtypes (IIa and IIb) alongside control specimens employed RNA sequencing followed by immunohistochemical validation with digital quantitative analysis (Assis-Mendonça et al., 2023). Among the differentially expressed genes, glycoprotein nonmetastatic melanoma protein B (GPNMB) emerged as one of the significantly upregulated transcripts in FCD IIb compared with both FCD IIa and control tissues. Immunohistochemical analyses demonstrated that GPNMB protein expression was almost exclusively confined to balloon cells within FCD IIb lesions (Assis-Mendonça et al., 2023). However, because some balloon cells were GPNMB-negative, GPNMB should be regarded as a balloon cell-associated marker rather than a universal marker of all balloon cells. GPNMB has previously been implicated in neurodegenerative diseases and cerebral ischemia–reperfusion injury, where it is associated with neuroprotection, modulation of inflammatory responses, and tissue repair processes (Hüttenrauch et al., 2018; Assis-Mendonça et al., 2023). Its selective enrichment in balloon cells raises the possibility that GPNMB participates in cellular stress responses, compensatory remodeling, or immunomodulatory signaling within the dysplastic cortical region (Hüttenrauch et al., 2018; Assis-Mendonça et al., 2023). This interpretation is compatible with key pathological features of FCD II, including abnormal neurotransmission, altered maintenance of neuronal membrane structures, and tissue inflammation (Assis-Mendonça et al., 2023), as well as the oxidative-stress and inflammatory milieu reported in FCD IIb lesions (Arena et al., 2019). Consequently, GPNMB has been proposed as a promising neuropathological biomarker associated with balloon cells. Nevertheless, validation in larger, multi-institutional cohorts will be required before incorporation into routine diagnostic practice.

Similarly, Avansini et al. investigated the role of neurogenin 2 (NEUROG2), a proneural transcription factor critical for mammalian neurogenesis, in FCD type II (Avansini et al., 2018). Their study revealed markedly elevated NEUROG2 expression in balloon cells and dysmorphic neurons, implicating aberrant activation of this neurogenic regulator in disease pathogenesis. NEUROG2, a member of the basic helix–loop–helix (bHLH) transcription factor family, plays a central role in cell fate determination and neuronal differentiation across multiple regions of the developing central nervous system (Hirabayashi and Gotoh, 2010). Under physiological conditions, NEUROG2 expression is tightly restricted to the ventricular zone and confined to the neurogenic phase, thereby coordinating the transition from neurogenesis to gliogenesis (Avansini et al., 2018). Dysregulation of NEUROG2 activity may therefore disrupt lineage commitment in neural progenitor populations, leading to ectopic or incompletely differentiated cell types. In FCD II, persistent or ectopic NEUROG2 expression may contribute to the emergence of dysmorphic neurons and transitional glioneuronal phenotypes such as balloon cells, reflecting arrested or aberrant differentiation trajectories (Avansini et al., 2018; Hirabayashi and Gotoh, 2010).

### 5.3 Pathogenic signaling pathways and functional effectors

Additional molecular studies have identified aberrant activation of signaling pathways that link developmental dysregulation to network hyperexcitability. One such example is fibroblast growth factor 13 (FGF13), which is significantly upregulated in FCD-associated cortical lesions relative to normal cortex. Immunohistochemical analyses demonstrate strong FGF13 expression in malformed cells, including dysmorphic neurons and the majority of balloon cells. Notably, FGF13 protein levels show a positive correlation with preoperative seizure frequency, suggesting a close association with disease severity and epileptogenicity (Wu et al., 2021). FGF13 belongs to the intracellular fibroblast growth factor (iFGF) family and functions as a microtubule-stabilizing protein involved in neuronal polarization, axon specification, and radial migration during cortical development. Aberrant upregulation of FGF13 may therefore disrupt microtubule dynamics, impair cytoskeletal organization, and interfere with orderly neuronal migration, contributing to the formation of dysmorphic neurons and balloon cells (Wu et al., 2012; Wu et al., 2021). In addition to its developmental roles, FGF13 may also influence neuronal excitability through the regulation of voltage-gated sodium channel localization and function. Prior studies have demonstrated that FGF13 interacts with voltage-gated sodium channels. Separately, increased Nav1.3 expression has been reported in FCD IIb lesions. Direct functional coupling between FGF13 and Nav1.3 in FCD tissue has not been demonstrated. The relevant channel partners and electrophysiological consequences of FGF13 upregulation therefore remain to be established, and it is also unclear whether FGF13 overexpression represents a primary pathogenic driver, a compensatory response, or an epiphenomenon secondary to mTOR dysregulation (Wu et al., 2021; Yu et al., 2012).

Recent research by Kumar et al. has highlighted histone deacetylase 6 (HDAC6) as another critical molecular effector in FCD II pathogenesis (Kumar et al., 2025). HDAC6, a predominantly cytoplasmic deacetylase, was found to be markedly upregulated in cortical tissues from FCD II patients and corresponding rat models. Functional analyses demonstrated that increased HDAC6 activity leads to reduced acetylation of peroxiredoxin 1 (Prdx1), a key cytoplasmic antioxidant enzyme. Because Prdx1 acetylation is required for efficient reactive oxygen species (ROS) scavenging, HDAC6-mediated deacetylation results in impaired antioxidant defense and elevated oxidative stress within dysplastic cortical tissue (Kumar et al., 2025). Inhibition of HDAC6 using the selective inhibitor Tubastatin A restored Prdx1 acetylation, reduced ROS accumulation, and significantly ameliorated epileptic phenotypes in FCD rat models, as evidenced by prolonged seizure latency and reduced seizure frequency (Kumar et al., 2025). These findings show that pharmacological inhibition of the HDAC6-Prdx1-oxidative stress pathway can improve seizure-related outcomes in an experimental FCD model (Kumar et al., 2025). Unlike expression or co-localization studies alone, this study provides direct pharmacological evidence supporting HDAC6 as a potential therapeutic target. However, because the intervention was tested at the tissue and animal-model level, these findings do not establish a BC-specific therapeutic mechanism.

## 6 Balloon cell heterogeneity and therapeutic stratification

Although balloon cells are a defining histopathological feature of FCD IIb, they should not be regarded as a uniform cell population with a single developmental origin or pathogenic function. Rather, available pathological, molecular, and spatial data indicate that BCs encompass partially overlapping abnormal cell states that vary in anatomical distribution, lineage commitment, marker expression, and interaction with the local epileptogenic microenvironment. Although BCs are morphologically recognized by enlarged cell bodies and immature cytological features, immunohistochemical studies indicate heterogeneous expression of progenitor-associated markers, including vimentin and nestin, as well as glial and neuronal markers such as GFAP, MAP2, and NeuN (Hadjivassiliou et al., 2010). These findings support the view that BCs represent developmentally arrested or lineage-ambiguous states rather than a homogeneous mature cell type.

This heterogeneity is also reflected in their proposed developmental origins. Depending on the timing and cellular context of somatic mTOR pathway mutations, abnormal cells may arise from multipotent radial glial progenitors, more lineage-restricted intermediate progenitors, or divergent developmental trajectories within the same lesion. Thus, BCs and DNs may share a common mutational origin in some lesions, whereas in others they may represent distinct phenotypic outcomes shaped by mutation timing, lineage context, and local microenvironmental cues. Spatial heterogeneity further complicates this picture, as BCs often intermingle with DNs and reactive glial populations within dysplastic lesions. Consistently, transcriptomic deconvolution and spatial transcriptomic studies indicate that FCD IIb lesions contain altered neuronal and glial compositions, including neuronal loss, reactive astrocyte signatures, and BC-enriched microenvironments associated with immune activation (Galvão et al., 2023; Zimmer et al., 2021; Wang et al., 2024).

Importantly, several markers proposed to characterize BCs are not uniformly expressed across all morphologically defined BCs. Markers such as GPNMB, adenosine kinase (ADK), and hyperpolarization-activated cyclic nucleotide-gated channel 4 (HCN4) further illustrate this heterogeneity, because each labels only selected BCs or mTOR-active abnormal cell populations rather than the entire morphologically defined BC compartment (Assis-Mendonça et al., 2023; Luan et al., 2015; Coleman et al., 2024). A similar caution applies to immune-related phenotypes. Immune-related signatures also appear heterogeneous and should be interpreted at the level of BC-enriched microenvironments rather than as uniform properties of all BCs (Zimmer et al., 2021; Wang et al., 2024). Therefore, it may be more accurate to describe specific BC subpopulations or BC-enriched microenvironments as contributors to inflammatory remodeling.

Recognizing BC heterogeneity has important implications for pathogenesis and therapy. Different studies have identified developmental, immune-related, ion-channel-associated, and purine-metabolism-associated features in BCs or BC-enriched regions. However, these observations were obtained using different cohorts and experimental approaches and should not be interpreted as evidence for established BC subtypes. Importantly, the available evidence differs among candidate molecules in both strength and cell-type specificity. Some findings are based on morphologically identified BCs or immunohistochemical co-localization, whereas others come from BC-enriched regions, whole FCD IIb tissue, or experimental models. Functional evidence also varies, from descriptive or correlative studies to pharmacological intervention in experimental FCD models. These differences are summarized in Table 1. Indeed, stable BC subtypes have not yet been defined at single-cell resolution. Nevertheless, this heterogeneity is critical for interpreting biomarker studies and therapeutic strategies. Accordingly, BC-directed therapeutic strategies should be interpreted with caution, because most candidate targets are unlikely to label or affect all BCs uniformly (Assis-Mendonça et al., 2023; Luan et al., 2015; Coleman et al., 2024; Zimmer et al., 2021; Wang et al., 2024). A key unresolved issue is whether these marker-defined populations correspond to stable BC subtypes, transient developmental states, or microenvironmentally induced phenotypes. Resolving this question will be essential for interpreting BC-directed biomarkers and therapies.

**TABLE 1:** Evidence levels and major limitations of balloon cell-associated molecular findings in FCD IIb.

Molecule/pathway	Main evidence source	Cellular specificity	Functional evidence	Major limitation/interpretation
GPNMB	Human FCD II transcriptomics with immunohistochemical validation (Assis-Mendonça et al., 2023)	Strongly enriched in morphologically identified BCs, although a subset of BCs is GPNMB-negative	No BC-specific genetic or pharmacological perturbation reported	A strong BC-associated histopathological marker, but its functional contribution to FCD IIb pathogenesis remains unresolved
ADK/adenosine system	Human FCD IIb immunohistochemistry and lesion-level biochemical analyses (Luan et al., 2015; Guo et al., 2023)	ADK detected in a large proportion of BCs; changes in ADA, CD73, A2AR and GLT-1 are largely lesion-level findings rather than BC-specific observations	No BC-specific intervention study in FCD	Supports disturbed purinergic homeostasis, but the causal contribution of BC-associated ADK to extracellular adenosine availability and seizure generation remains untested
HCN4	Human mTORopathy/FCD II tissue with immunohistochemical and immunofluorescence analyses (Coleman et al., 2024)	Enriched in p-S6-positive abnormal cells, including DNs and BCs; not specific to BCs	No HCN4-targeted intervention demonstrated in an FCD model	Represents a candidate biomarker and exploratory therapeutic target; a BC-specific functional role has not been established
LILRB2 pathway	Human FCD IIb cortical tissue; expression and co-localization analyses (Yue et al., 2019)	Detected in neuronal and astrocytic compartments and not restricted to BCs	Correlative evidence; no cell-specific functional intervention	The inverse association with seizure frequency argues against a simple pro-epileptogenic interpretation; effects may be compensatory or context dependent
CB1/endocannabinoid system	Human FCD II tissue with confocal microscopy and molecular analyses (García-Rincón et al., 2018)	Predominantly localized to p-S6-positive dysplastic cells, including BCs and other abnormal cell populations	Pharmacological CB1 blockade attenuated mTORC1 overactivation in FCD-derived organotypic cultures; no BC-specific or seizure-outcome intervention	Provides functional pathway evidence at the *ex vivo* lesion level, but does not establish BC-specific CB1 signaling as a causal driver of seizures
CD47/SIRP-α	Human FCD IIb lesion analyses and immunohistochemistry (Sun et al., 2016)	Altered in abnormal cells and neuroimmune compartments; not exclusively BC-specific	Limited lesion-level mechanistic evidence; no BC-selective perturbation	Potentially relevant to neurite development and microglial regulation, but the specific contribution of BCs cannot be separated from broader lesion effects
Plexin-B2/Sema4C	Human FCD IIb tissue with expression and histopathological analyses (Dai et al., 2023; Dai et al., 2024)	Abnormal expression reported in BCs and DNs, with heterogeneous cellular distribution	Primarily correlative; no BC-specific functional intervention	May reflect abnormal developmental or synaptic states, but a direct contribution to BC-mediated epileptogenesis remains unresolved
FGF13	Human FCD tissue with immunohistochemical analyses and clinicopathological correlation (Wu et al., 2021)	Expressed in malformed cells, including DNs and many BCs; not BC-specific	No FCD-specific cell-type-selective perturbation	Association with seizure frequency does not establish causality; whether FGF13 is pathogenic, compensatory, or secondary to mTOR dysregulation remains unclear
HDAC6-Prdx1	Human FCD II tissue and experimental FCD rat model (Kumar et al., 2025)	Demonstrated at tissue/model level rather than specifically in BCs	Pharmacological HDAC6 inhibition reduced oxidative stress and improved seizure phenotypes in an experimental FCD model	Provides comparatively stronger functional evidence for an FCD-associated therapeutic mechanism, but does not establish a BC-directed effect
Complement/immune signaling	Human FCD IIb bulk and spatial transcriptomic analyses with histological validation (Zimmer et al., 2021; Wang et al., 2024; Rossini et al., 2023)	Predominantly derived from BC-enriched regions and surrounding microglial/astrocytic responses; selected complement-associated proteins have also been localized to BCs	Lesion-level evidence links complement activation to synaptic remodeling; no BC-selective causal perturbation	BC-enriched regions are strongly associated with immune and complement activation, but whether BCs initiate, sustain, or merely coexist with these responses remains unresolved

A2AR, adenosine A2A receptor; ADA, adenosine deaminase; ADK, adenosine kinase; BC, balloon cell; CB1, cannabinoid type 1 receptor; CD47, cluster of differentiation 47; DN, dysmorphic neuron; FCD, focal cortical dysplasia; FGF13, fibroblast growth factor 13; GLT-1, glutamate transporter 1; GPNMB, glycoprotein nonmetastatic melanoma protein B; HCN4, hyperpolarization-activated cyclic nucleotide-gated channel 4; HDAC6, histone deacetylase 6; LILRB2, leukocyte immunoglobulin-like receptor subfamily B member 2; mTOR, mechanistic target of rapamycin; Plexin-B2, plexin B2; Prdx1, peroxiredoxin 1; p-S6, phosphorylated ribosomal protein S6; Sema4C, semaphorin 4C; SIRP-α, signal regulatory protein α.

## 7 Therapeutic implications of balloon cell-associated pathways

Epilepsy surgery remains the most effective intervention for achieving sustained seizure control and preventing progressive neurodevelopmental impairment in patients with FCD (Catchpool et al., 2019). However, its clinical applicability is limited by several factors. Surgical candidacy is often constrained when the epileptogenic zone involves eloquent cortical regions or when lesions are extensive, posing unacceptable risks to neurological function. Moreover, the invasiveness, substantial economic burden, and potential perioperative complications associated with neurosurgical procedures highlight the need to develop effective nonsurgical therapeutic alternatives. Accordingly, the identification of novel, mechanism-based therapeutic targets capable of suppressing seizures while preserving neurological integrity remains clinically important. Importantly, the evidence supporting these candidate therapeutic pathways varies considerably. Some findings are based mainly on altered expression or cellular co-localization in human FCD tissue, whereas others have been tested using pharmacological or genetic interventions in experimental models. None of the studies discussed below has yet established a BC-specific therapeutic target in FCD IIb. We therefore distinguish expression-based candidates from pathways that have been tested functionally.

### 7.1 HCN4 channels

Recent mechanistic studies have identified aberrant expression of HCN4 in FCD tissues derived from patients with mTORopathies of diverse genetic etiologies. These findings suggest that HCN4 dysregulation may constitute a convergent downstream consequence of pathological mTOR signaling. Histopathological analyses have demonstrated markedly elevated steady-state levels of HCN4 within FCD type II lesions, despite considerable variability in variant allele frequencies across specimens. Notably, immunofluorescence studies revealed robust co-localization of p-S6, a canonical marker of mTORC1 activation, with HCN4 expression, indicating that HCN4 upregulation is largely confined to cell populations exhibiting heightened mTOR activity (Coleman et al., 2024). This spatial convergence supports the interpretation that dysregulated HCN4 expression is preferentially associated with dysmorphic neurons and balloon cells, but does not establish a BC-specific functional role for HCN4 in epileptogenesis (Coleman et al., 2024). Functionally, HCN4 channels can modulate membrane potential and neuronal excitability, providing a plausible mechanism by which ectopic HCN4 expression could influence dysplastic neuronal networks (Kharouf et al., 2020; Coleman et al., 2024). However, the current evidence in FCD is primarily based on expression and cellular localization, and direct pharmacological or genetic intervention targeting HCN4 has not yet been demonstrated to alter seizure phenotypes in an FCD model. HCN4 should therefore be considered an exploratory therapeutic candidate rather than an established BC-directed therapeutic target.

### 7.2 Dysregulation of the adenosine system

Developmental lineage analyses have revealed prominent immunoreactivity of ADK in balloon cells within FCD type II lesions, with approximately 77% of BCs exhibiting ADK expression and considerable heterogeneity across cell populations expressing different lineage markers. Consistently, ADK levels are markedly elevated in FCD specimens (Luan et al., 2015), identifying increased ADK expression as a recurring pathological feature of FCD type IIb. As ADK serves as the principal enzyme mediating intracellular adenosine clearance, its overexpression is predicted to reduce extracellular adenosine availability, thereby compromising one of the brain’s most potent endogenous anticonvulsant mechanisms. Subsequent studies further demonstrated that both FCD type I and type II lesions exhibit upregulated expression of multiple adenosine-metabolizing enzymes, including ADK and adenosine deaminase (ADA), as well as the synaptic adenosine-generating enzyme CD73, collectively indicating accelerated adenosine turnover and disrupted purinergic homeostasis. Additionally, immunoreactivity for the adenosine A2A receptor (A2AR) is markedly increased, potentially exacerbating aberrant downstream signaling in response to altered adenosine dynamics. In FCD specimens, Guo et al. reported reduced GLT-1 levels together with increased mTOR levels relative to control tissue (Guo et al., 2023). These findings reflect abnormalities within the FCD lesion and should not be interpreted as evidence that BCs themselves uniformly exhibit reduced GLT-1 expression. Reduced GLT-1 availability may impair astrocyte-mediated glutamate clearance and contribute to extracellular glutamate accumulation within the dysplastic tissue. The relationship between purinergic dysregulation, glutamate transport, and mTOR signaling requires cell-type-resolved investigation. Together, these findings suggest that abnormal adenosine metabolism may represent a potential therapeutic target in FCD-related epilepsy. However, the available evidence is based mainly on expression and lesion-level analyses, and it remains unclear whether BC-associated ADK expression directly alters adenosine homeostasis or seizure generation. The adenosine system therefore requires further functional and cell-type-specific studies before its therapeutic role in FCD can be defined.

### 7.3 Endocannabinoid system alterations

Emerging evidence also implicates dysregulation of the endocannabinoid system in the pathophysiology of FCD. Garcia-Rincon et al. demonstrated that cannabinoid type 1 receptor (CB1) signaling regulates neurodevelopmental processes, at least in part, through modulation of mTORC1 activity. Using confocal microscopy, they characterized CB1 receptor expression and mTORC1 activation in surgically resected tissue from patients with FCD type II (García-Rincón et al., 2018). Genetic screening of endocannabinoid system components, combined with CB1 receptor gene sequencing, revealed that CB1 receptors were predominantly localized to p-S6-positive cells, including balloon cells, which co-expressed aberrant markers associated with undifferentiated progenitors and dysplastic neurons. These observations show an association between CB1 expression and mTOR-active dysplastic cells, including BCs, but do not demonstrate that CB1 signaling within BCs directly affects neuronal excitability or seizure generation (García-Rincón et al., 2018). The endocannabinoid pathway should therefore be regarded as a potential therapeutic target that requires further functional study in FCD II. Direct studies using cell-type-specific manipulation will be needed to determine whether CB1 signaling contributes to epileptogenesis and whether targeting this pathway has therapeutic value.

### 7.4 Selective ablation strategies

Beyond pharmacological modulation, selective ablation has been experimentally tested to determine whether specific dysplastic cells drive epileptogenesis in FCD type II. Ying Xu et al. tested this concept by introducing the diphtheria toxin receptor (DTR) specifically into dysmorphic neurons in a validated FCD type II mouse model, followed by systemic administration of diphtheria toxin to induce targeted cell ablation. Remarkably, DTR-mediated ablation, whether implemented preventively prior to seizure onset or therapeutically after seizure establishment, completely abolished both spontaneous and pentylenetetrazole-induced seizures in this model. This intervention also reversed reactive gliosis and normalized aberrant neuronal excitability in adjacent cortical regions, suggesting restoration of local network homeostasis. Compared with rapamycin treatment, selective cell ablation conferred durable seizure suppression, with no seizure recurrence after treatment cessation and without the need for continuous systemic mTOR inhibition. However, the Xu et al. study targeted dysmorphic neurons rather than human balloon cells, and it remains unknown whether BCs would respond similarly to ablation-based approaches. Moreover, current *in vivo* models do not fully recapitulate the molecular identity, morphology, or heterogeneity of human BCs. Therefore, the therapeutic significance of selective ablation of balloon cells in FCD II remains speculative and requires further validation. This contrast highlights an important evidence gap: causal cell-selective intervention data are available for DNs in an experimental FCD model, whereas comparable evidence demonstrating that manipulation of BCs alters seizure generation or maintenance is currently lacking.

## 8 Conclusion and future perspectives

FCD II represents one of the most common pathological substrates underlying medically intractable epilepsy in children. Among its defining histopathological features, balloon cells have emerged as a central element for understanding disease mechanisms and identifying novel therapeutic opportunities. This review synthesizes current advances in elucidating the developmental origin, molecular phenotype, electrophysiological characteristics, inflammatory functions, and network-level impact of balloon cells, outlining a multifaceted pathological framework shaped by aberrant mTOR signaling, neuroimmune activation, epigenetic dysregulation, and oxidative stress. Balloon cells are a morphologically defined but molecularly heterogeneous component of FCD IIb lesions. Current evidence links BC-enriched lesions to developmental abnormalities, altered cellular composition, and immune or complement-related remodeling; however, it does not yet distinguish BC-intrinsic causal mechanisms from changes induced by dysmorphic neurons, glia, or the broader lesion microenvironment.

Several specific experimental questions now need to be addressed. First, single-cell or single-nucleus multi-omic profiling combined with spatial and morphological validation could determine whether GPNMB-, ADK-, or HCN4-positive BC populations represent stable molecular states, transient developmental phenotypes, or changes induced by the local microenvironment. Second, integrating cell-type-resolved somatic genotyping with transcriptomic profiling could more systematically define how pathogenic mTOR-pathway variants are distributed across BCs, DNs, and other cell populations and how genotype relates to cellular phenotype. Third, because many immune and complement-related signatures have been identified at the level of BC-enriched regions or whole lesions rather than purified BCs, spatially resolved and cell-type-specific approaches will be required to distinguish BC-intrinsic signaling from responses of surrounding microglia, astrocytes, and other cells. Finally, human organoid models combined with lineage tracing and cell-type-specific genetic or functional perturbation could directly test whether the formation, manipulation, or removal of BC-like cells alters neighboring neuronal maturation, synaptic organization, or network excitability. Together, these approaches should help determine whether BCs act as causal effectors, indirect modulators of the dysplastic microenvironment, or primarily histopathological manifestations of the underlying developmental lesion.

## Glossary

4EBP1: Eukaryotic translation initiation factor 4E-binding protein 1
A2AR: Adenosine A2A receptor
ADA: Adenosine deaminase
ADK: Adenosine kinase
AKT: Protein kinase B
BC/BCs: Balloon cell(s)
Bcl-2: B-cell lymphoma 2
CB1: Cannabinoid type 1 receptor
CCL2: C-C motif chemokine ligand 2
CD34: Cluster of differentiation 34
CD47: Cluster of differentiation 47
CD133: Cluster of differentiation 133
CNS: Central nervous system
DN/DNs: Dysmorphic neuron(s)
DT: Diphtheria toxin
DTR: Diphtheria toxin receptor
FCD: Focal cortical dysplasia
FGF13: Fibroblast growth factor 13
GATOR1: GAP activity toward Rags 1
GFAPδ: Glial fibrillary acidic protein δ
GPNMB: Glycoprotein nonmetastatic melanoma protein B
HCN4: Hyperpolarization-activated cyclic nucleotide-gated channel 4
HDAC6: Histone deacetylase 6
HLA-II: Human leukocyte antigen class II
iFGF: Intracellular fibroblast growth factor
ILAE: International League Against Epilepsy
IPCs: Intermediate progenitor cells
Klf4: Krüppel-like factor 4
LILRB2: Leukocyte immunoglobulin-like receptor subfamily B member 2
Mcm2: Minichromosome maintenance complex component 2
mIPSC: Miniature inhibitory postsynaptic current
MRI: Magnetic resonance imaging
mTOR: Mechanistic target of rapamycin
mTORC1: Mechanistic target of rapamycin complex 1
Nav1.3: Voltage-gated sodium channel 1.3
NEUROG2: Neurogenin 2
PCNA: Proliferating cell nuclear antigen
PI3K: Phosphoinositide 3-kinase
Prdx1: Peroxiredoxin 1
p-S6: Phosphorylated ribosomal protein S6
PTEN: Phosphatase and tensin homolog
PV: Parvalbumin
REDs: Recurrent epileptiform discharges
RGPs: Radial glial progenitors
RHEB: Ras homolog enriched in brain
RNA-seq: RNA sequencing
ROS: Reactive oxygen species
S6K1: S6 kinase 1
Sema4C: Semaphorin 4C
SIRP-α: Signal regulatory protein α
Sox2: SRY-box transcription factor 2
TSC: Tuberous sclerosis complex
Tuba: Tubastatin A
VEGF-C: Vascular endothelial growth factor C
